# Genome-Wide Assessment of Runs of Homozygosity in Chinese Wagyu Beef Cattle

**DOI:** 10.3390/ani10081425

**Published:** 2020-08-14

**Authors:** Guoyao Zhao, Tianliu Zhang, Yuqiang Liu, Zezhao Wang, Lei Xu, Bo Zhu, Xue Gao, Lupei Zhang, Huijiang Gao, George E. Liu, Junya Li, Lingyang Xu

**Affiliations:** 1 Key Laboratory of Animal Genetics Breeding and Reproduction, Ministry of Agriculture and Rural Affairs, Institute of Animal Science, Chinese Academy of Agricultural Sciences, Beijing 100193, China.; zhaoyifan687@163.com (G.Z.); zhangtianliu92@foxmail.com (T.Z.); yuqiangliu123@126.com (Y.L.); wangzezhao1@163.com (Z.W.); xuleirock@163.com (L.X.); zhubo525@126.com (B.Z.); gaoxue76@126.com (X.G.); zhanglupei@caas.cn (L.Z.); gaohj111@sina.com (H.G.); 2 National Engineering Laboratory for Animal Breeding, Key Laboratory of Animal Genetics, Breeding and Reproduction, Ministry of Agriculture, College of Animal Science and Technology, Chinese agriculture University, Beijing 100193, China; 3 Animal Genomics and Improvement Laboratory, United States Department of Agriculture-Agricultural Research Service, Beltsville, MD 20705, USA; george.liu@usda.gov

**Keywords:** runs of homozygosity, inbreeding level, consensus ROH, important traits, Chinese Wagyu beef cattle

## Abstract

**Simple Summary:**

Runs of homozygosity (ROH) are continuous homozygous regions that generally exist in the DNA sequence of diploid organisms. Identifications of ROH leading to reduction in performance can provide valuable insight into understanding the genetic architecture of complex traits. We assessed genome-wide homozygosity patterns and inbreeding levels in Chinese Wagyu beef cattle. We identified several high frequency regions overlapped with QTLs which are associated with body weight, calving ease, and stillbirth. Based on these regions, we carried out a region-based association for eight economically important traits. Of these, nine regions were significantly associated with six traits including body height, chest circumference, fat coverage, backfat thickness, ribeye area, and carcass length. Our study identified that many candidate regions and genes overlapped with ROH for several important traits, which could be unitized to assist the design of a selection mating strategy in beef cattle.

**Abstract:**

Runs of homozygosity (ROH) are continuous homozygous regions that generally exist in the DNA sequence of diploid organisms. Identifications of ROH leading to reduction in performance can provide valuable insight into the genetic architecture of complex traits. Here, we evaluated genome-wide patterns of homozygosity and their association with important traits in Chinese Wagyu beef cattle. We identified a total of 29,271 ROH segments from 462 animals. Within each animal, an average number of ROH was 63.36 while an average length was 62.19 Mb. To evaluate the enrichment of ROH across genomes, we initially identified 280 ROH regions by merging ROH events across all individuals. Of these, nine regions containing 154 candidate genes, were significantly associated with six traits (body height, chest circumference, fat coverage, backfat thickness, ribeye area, and carcass length; *p* < 0.01). Moreover, we found 26 consensus ROH regions with frequencies exceeding 10%, and several regions overlapped with QTLs, which are associated with body weight, calving ease, and stillbirth. Among them, we observed 41 candidate genes, including *BCKDHB*, *MAB21L1*, *SLC2A13*, *FGFR3*, *FGFRL1*, *CPLX1*, *CTNNA1*, *CORT*, *CTNNBIP1*, and *NMNAT1*, which have been previously reported to be related to body conformation, meat quality, susceptibility, and reproductive traits. In summary, we assessed genome-wide autozygosity patterns and inbreeding levels in Chinese Wagyu beef cattle. Our study identified many candidate regions and genes overlapped with ROH for several important traits, which could be unitized to assist the design of a selection mating strategy in beef cattle.

## 1. Introduction

Runs of homozygosity (ROH) are continuous homozygous regions that generally exist in the DNA sequence of diploid organisms [1]. Several studies have suggested that an individual inherits the chromosomal fragment from both parents, resulting in homozygous segments in the offspring’s genome [2,3]. Many factors can contribute to the formation of ROH, including paternal founder effects, genetic drift, inbreeding, and intensive selection [4]. Long ROH are most likely derived from a recent ancestor while shorter ones are from a more distant ancestor [5].

With the development of high-density array and whole-genome sequencing technologies, they provided unprecedented opportunities to explore homozygosity segments in high resolution [6].

Initially, Broman et al. proposed that ROH exist widely in the human genome and has an important impact on human health [7]. Jane et al. further explored different distribution frequencies of ROH with various lengths using high-density SNP arrays, promoting the development for the ROH study in humans [1]. Additional analyses found that ROH can provide insights into the population structure and demography in humans [8,9,10] and genetic relationships [11,12,13,14,15,16]. The enrichment of ROH may increase the harmful recessive alleles, and reduce the survival ability of individuals [17]. Several papers have also found a significant association between ROH and the occurrence of recessive diseases [18,19]. Other investigations suggested that ROH were related to human diseases, including schizophrenia [20], rheumatoid arthritis [21], and Alzheimer’s disease [22].

In recent years, ROH have been widely utilized to assess the genome-wide inbreeding level and genetic relationship in farm animals [11,15,23,24,25,26,27,28,29,30,31]. ROH can provide a novel perspective for detecting detrimental variations in the genome by assessing the inbreeding level and offering important information of genetic and demographic history [32].

In cattle, Ferencakovic et al. investigated genome-wide ROH patterns and revealed that inbreeding coefficients calculated by ROH were useful for assessing inbreeding levels [11]. Moreover, a more accurate estimation based on ROH can be obtained for inbreeding coefficients, when compared with the pedigree data [28]. Moreover, ROH can reflect the genetic relationship and inbreeding levels among groups, and affect the selection on the genomic region in cattle [30,33]. Previous studies suggested that the formation of ROH may involve the selection and some regions of ROH are related to economical important traits in farm animals [31,34,35]. The Chinese Wagyu population has been generated between Wagyu cattle and Fuzhou local cattle in China. It is well-known for its meat quality including beef flavor and tenderness. Based on this population, we have previously identified many candidate genes associated with body measurement traits and fatty acid composition [36,37]. However, there is no systematic assessment of ROH patterns and their association with important traits in Chinese Wagyu beef cattle. The aims of this study were to (i) assess genome-wide ROH patterns and inbreeding levels using a high density array; (ii) characterize profiles of ROH with different sizes and the gene contents with ROH hotspots; and (iii) identify potential ROH regions associated with economically important traits in the Chinese Wagyu beef cattle population.

## 2. Materials and Methods

### 2.1. Genotyped Samples

The BovineHD chip data of Chinese Wagyu beef cattle was extracted from our previous publication [36]. The population was established in Dalian XueLong Co. Ltd., Liaoning Province, China. Only autosomal SNP markers were analyzed for subsequent analysis. Then, individuals with missing SNPs exceeding 5% were removed. The PLINK v1.90 software was used to check the quality of the dataset [38], and quality control standards were set as follows: Minor allele frequency (MAF) > 0.05, genotype missing rate < 10%. After quality control, 503,579 SNPs from 462 individuals were used for subsequent analyses.

### 2.2. ROH Estimation

In this study, we used PLINK v1.90 to detect ROH on the autosomes for each individual. Since the linkage disequilibrium (LD) can cause short and common ROH throughout the genome, ROH were defined to be at least 0.5 Mb in this study. The specific parameters were as follows: 50 SNPs sliding windows slid along the chromosome to detect homozygous segments in each individual, and the sliding window allowed no more than one heterozygote. Several important parameters of defining ROH are involved: (1) The minimum length was 500 kb; (2) the proportion of homozygous overlap window was 0.05; (3) the minimum number of consecutive SNPs included in a ROH was 100; (4) the minimum SNP density was set to 50 kb/SNP; (5) the maximum gap between continuous homozygous SNPs was 100 kb; and (6) a maximum of two SNPs with missing genotypes and up to one heterozygous genotype were allowed in a ROH.

### 2.3. ROH Classification and Inbreeding Coefficients

The length of ROH were divided into three classes: Small (0.5−1 Mb), Medium (1−5 Mb), or Large (>5 Mb). Two methods were used to calculate the inbreeding coefficient for each individual, including: (1) F_HOM_ was assessed based on the proportion of homozygotes using PLINK v1.90; (2) F_ROH_ was calculated as described by McQuillan et al., i.e., an individual’s summed ROH length was normalized by the length of the autosomal genome covered by SNPs [39]. Correlations of the inbreeding coefficient for two methods were estimated using the *cor.test* function in R v3.2.4 (https://www.r-project.org/).

### 2.4. QTL Regions and Genomic Regions within ROH

To explore the potential QTL related to economic traits and diseases, we adopted an approach plugged in PLINK v1.90 (http://www.cog-genomics.org/plink/1.9/) [38,40] (–homozyg -group) to estimate the consensus regions across individuals, which represent ROH pools of overlapping and potentially matching segments. Then, based on these consensus regions, we annotated QTL and reference genes, based on QTLdb and the UMD3.1 genome assembly, respectively, when ROH frequencies exceeded 10%. Function annotations of the identified genes and GO terms were further assessed using the DAVID platform [41,42].

### 2.5. Region Association

ROH regions were generated by aggregating overlapping CNVs (by at least 1 bp) across samples using Bedtools v2.26.0 (https://bedtools.readthedocs.io/en/latest/) [43]. The proportion of ROH coverage for each region within an individual were considered as variable. The raw phenotype was adjusted by the general linear model. The fixed effects included farm, age, gender, and generation. Eight traits, including fat coverage, slaughter weight, backfat thickness, ribeye area, carcass length, body height, chest circumference, and body length were utilized for analyses. Association analyses between ROH region and the adjusted phenotype were conducted using a linear regression, and candidate regions were considered statistically significant when *p* < 0.01. Accordingly, a multiple correction was carried out for each region using the q-value package [44], and the suggestive threshold for multiple tests was set to 0.1. All statistical analyses were carried out using R version 3.2.4 (https://www.r-project.org/).

### 2.6. Ethics Statement

An ethics statement was not required for this study. The dataset from the animals included in this study were from previous analyses that obtained specific permissions [36].

## 3. Results

### 3.1. Genomic ROH Distribution

We identified a total of 29,271 ROH in 462 Chinese Wagyu beef cattle. The longest ROH were identified on BTA10 (48.6 Mb with 10,015 SNPs), while the shortest ROH were detected on BTA3 (0.5 Mb with 130 SNPs). We found that most of the ROH (98.50%) were relatively short. In addition, we observed that the number of ROH varied across chromosomes, with the highest number of ROH on BTA1 (2463 ROH segments) and the minimum number of ROH on BTA25. In each animal, we identified an average number of 63.36 ROH segments, with an average length of 62.19 Mb, covering a relatively small portion of the genome (Supplementary Materials Figure S1).

### 3.2. ROH Region and Inbreeding Coefficients

ROH regions were generated by merging ROH identified across all individuals using previously published protocols, implemented in Bedtools [43]. We identified 280 nonredundant ROH regions in 462 individuals. Then, we estimated inbreeding coefficients of the studied population using two different approaches, including F_HOM_ and F_ROH_. The inbreeding coefficient of F_HOM_ varied from −0.13 to 0.27, and the values of F_ROH_ varied from 0.01 to 0.33. Our results also showed that F_HOM_ and F_ROH_ were highly correlated (*r* = 0.96, *p* < 2.2 × 10^−6^).

### 3.3. Genomic Patterns of Homozygosity

**Figure 1:**
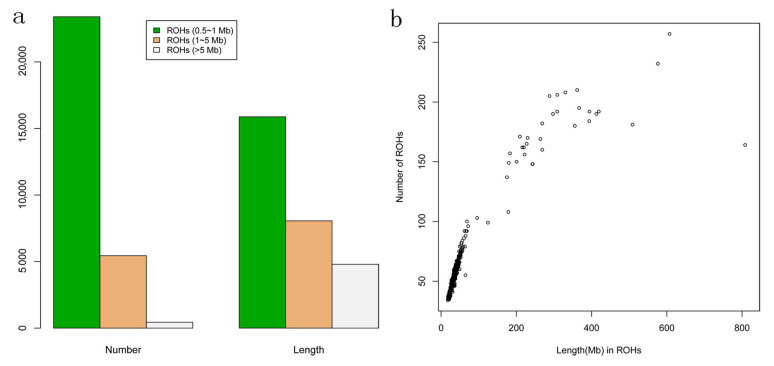
(**a**) The total numbers and lengths of runs of homozygosity (ROH) of three classes including Small (0.5 to 1 Mb), Medium (1 to 5 Mb), and Large (>5 Mb) classes. (**b**) Correlation of total ROH numbers and lengths. The ROH number (*y*-axis) is plotted against the ROH length (*x*-axis, i.e., the length of Mb covered by ROH) in each individual.

To evaluate the genomic pattern of ROH, we divided the length of ROH into three size classes: a. Small (500 kb to 1 Mb), b. Medium (1 to 5 Mb), or c. Large (>5 Mb), as described in the previous study [8]. The ROH distributions in terms of total number and length were shown in Figure 1a. We observed a total of 23,390 Small (500 kb to 1 Mb) with a total length of 15,876.92 Mb, while a total of 440 Large (>5 Mb) with a total length of 4797.37 Mb. Moreover, we found that the total lengths of ROH and the numbers of ROH were highly correlated (*r* = 0.91, *p* < 2.2 × 10^−6^), and the numbers of ROH were positively correlated with the lengths of autosomes (Figure 1b).

### 3.4. The Consensus of ROH within the Population

**Figure 2:**
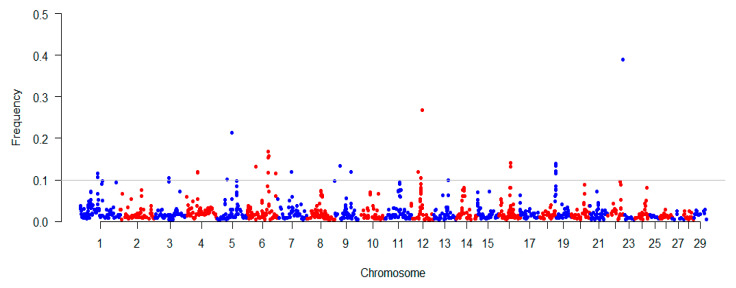
Distribution characteristics of ROH on chromosomes in Chinese Wagyu beef cattle. The horizontal axis is the single nucleotide polymorphism (SNP) position, which is ordered by the physical location of the genome; the vertical axis is the ROH frequency at this SNP. The horizontal line in the graph is where the ROH frequency equals 10%.

To explore the occurrence of ROH within our studied population, we estimated the consensus ROH (common ROH among the population) using the PLINK command (-homozyg -group) across the genome. We found that the distribution of consensus of ROH among different chromosomes was uneven (Figure 2). For instance, the highest frequency of ROH (~39%) was observed in the middle part of BTA23. We also found that two ROH regions with frequencies of ~26% and ~21% were located at BTA12 and BTA5, respectively. We totally detected 26 regions with the ROH frequency exceeding 10% among the studied population, and these regions overlapped with 41 RefGenes based on UMD 3.1 (Supplementary Materials Table S1). Additionally, based on the cattle QTLdb, 35 QTLs were found to be overlapped with 26 regions. Notably, we found that the high frequency regions were overlapped with QTLs, which are associated with body weight (at BTA4) and calving ease (at BTA6 and BTA23). Moreover, it was noted that these overlaps included several important QTLs for traits including gestation length (at BTA5), lean meat yield (at BTA12), and stillbirth (at BTA23) (Table 1). Among the detected 26 regions with the ROH frequency exceeding 10%, we observed 41 RefGenes, and these genes including *BCKDHB*, *MAB21L1*, *SLC2A13*, *FGFR3*, *FGFRL1*, *CPLX1*, *CTNNA1*, *CORT*, *CTNNBIP1*, and *NMNAT1*, which have been previously reported are related to several important traits including body conformation, meat quality, susceptibility, and reproductive efficiency in cattle.

### 3.5. ROH Region-Based Association Analysis

**Figure 3:**
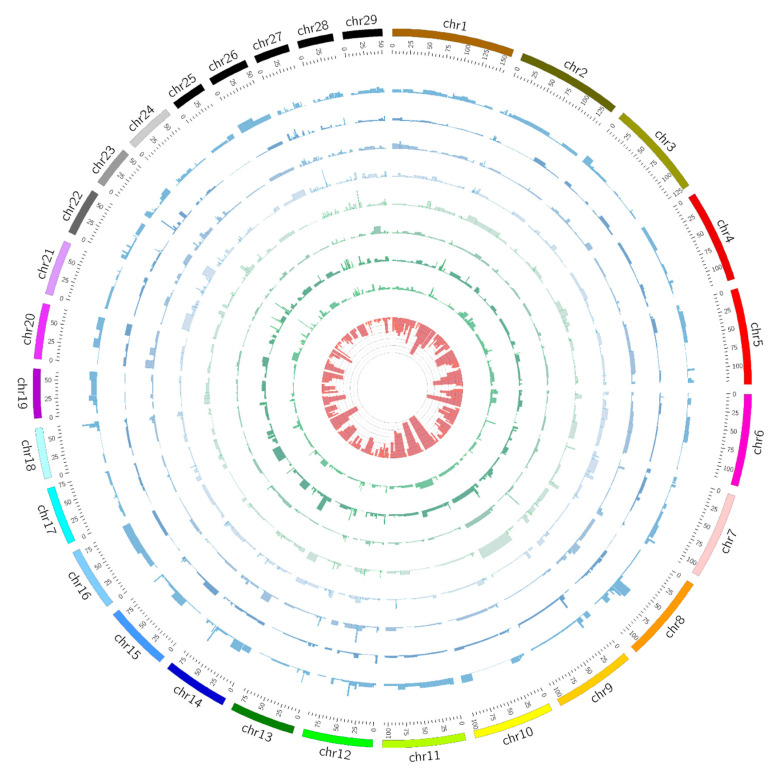
Circos plot illustrating ROH regions and association results for eight traits. Association significance test for each of ROH was plotted based on −log_10_ (*p*) values using histograms within the gray inner circle. The ROH frequency was determined by occurrence of ROH with each region within the population. The estimated frequency values were shown in the innermost circle. The outermost circle displays the cattle autosomes. The circles from inside to outside represent association results for body height, body length, chest circumference, fat coverage, backfat thickness, ribeye area, carcass length, and slaughter weight, respectively.

We totally identified 280 regions and performed an association analysis using a linear model for eight economical important traits. The summary statistics of ROH regions were presented in (Supplementary Materials Table S2). We generated a genome-wide ROH region plot using Circos (http://circos.ca/), which illustrated ROH regions and association results for eight traits (Figure 3). For body height, two significant ROHs were found at BTA23 and BTA7, which in turn spanned approximately 1.2 and 3.6 Mb, covering 34 and 56 candidate genes. Moreover, we identified one significant ROH (*p* < 0.01) for the chest circumference (BTA8, 1.9 Mb, containing 33 candidate genes), the backfat thickness (BTA27, 0.6 Mb, 22 genes), the ribeye area (BTA12, 2.1 Mb, no gene), and the fat coverage (BTA28, 1 Mb, 4 genes), respectively. In addition, we detected three significant ROH regions at BTA9, BTA23, and BTA27 for the carcass length. Among them, three regions pass the suggestive threshold (*q*-value < 0.1) after multiple tests with the false discovery rate (FDR). In Table 2, we presented the genomic coordinates of ROH regions and the numbers of their overlapped genes for important traits using a region-based association analysis.

## 4. Discussion

Many studies have explored the genome-wide ROH pattern and inbreeding depression in cattle populations [3,23,45]. Our study attempted to investigate the occurrence and distribution of ROH in Chinese Wagyu beef cattle using the high-density SNP array. A previous study suggested that high-density SNP arrays are more sensitive to determine small segments, thus Bovine SNP50K arrays may underestimate the number of fragments with the length of 1−4 Mb [46]. In the present study, our results showed that the most abundant ROH segments ranged from 0.5 to 1 Mb, which implied the power of high-density SNP arrays for identification of small ROH.

We estimated inbreeding coefficients using two methods, including F_ROH_ and F_HOM_. In general, the correlation between F_ROH_ and F_HOM_ varies across studies. A previous study found that the estimated correlation ranged from 0.78 to 0.85, and F_ROH_ has a high correlation with F_HOM_, thus F_ROH_ can be used as an accurate estimate of the proportion of IBD (identity by descent) genomes [47]. In this study, the significant correlation (*r* = 0.96) was observed between F_ROH_ and F_HOM_, which is consistent with the previous report [48].

Many studies have shown significant differences in the total numbers and lengths of ROH in cattle, as well as the genetic relationships between individuals [9,49]. The distribution patterns of ROH count and length may indicate the differences in breed formation and recent breed management. For instance, analysis of the British Isles breeds including Hereford, Guernsey, Angus, and Jersey cattle displayed the highest total number of ROH events per animal when compared to other breeds, while African breeds displayed high variability in the total number of ROH among breeds [9]. As described by a previous study, the average total length of ROH was 106 and 371 Mb for Piedmontese and Brown cattle, and the ROH identified in dairy breeds was longer than those in beef and dual-purpose cattle [50]. Several studies suggest that the short ROH reflects ancient inbreeding, and the long ROH may reveal recent inbreeding [32,50]. Most ROH identified in our study belonged to the Short and Medium classes, and this indicated that the Chinese Wagyu population has a low inbreeding level, which was in line with its population history, i.e., this population has undergone a recent hybrid process. Since the SNP array is still limited by its probe density and non-uniform distribution across the genome, the ascertainment biases limit a formal statistical testing of the evidence for the selection of ROH. In addition, it should be noted that population bottlenecks and genetic drift can also affect the formation of ROH [8]. Moreover, a previous simulation study has tested out ROH calling programs and optimal settings [6], the ROH calling methods and their settings are still challenging [9,46]. Moreover, to avoid bias and overestimation for ROH detection using PLINK, more options should be considered carefully according to the genome coverage and the density of SNPs array [51].

Our study identified many consensuses of ROH in the Chinese Wagyu population, and the distribution of ROH across genomic regions may indicate a potential selection effect for traits. We detected 26 regions with ROH frequency exceeding 10%. Of these, we identified several genes to be related to body conformation, meat quality, susceptibility, and reproductive efficiency. For instance, *BCKDHB* was found to be related to the attachment width in Chinese Holstein cattle [52], and this gene was also reported to display a significant expression associated with RFI in Australian Angus cattle [53]. *MAB21L1* were detected with a higher expression in *longissimus lumborum* and *psoas major* [54], which may result in distinct metabolic patterns and physiological processes for meat quality. Notably, *SLC2A13* have been implicated in susceptibility to the *Mycobacterium avium ssp. paratuberculosis* tissue infection [55] and resistance to clinical mastitis [56]. Moreover, we observed two genes (*FGFR3* and *FGFRL1*), which were identified as negative regulators of endochondral ossification [57] and body size [58]. Several genes, including *CTNNA1*, *LRRTM2*, *SIL1*, and *NMNAT1*, have been implicated under the selection [59,60,61,62] and overlapped with the previously identified regions of ROH [49,63]. We also found two genes (*CTNNBIP1* and *NMNAT1*) to be related to reproductive efficiency. *CTNNBIP1* has been previously reported to be related to prenatal death in Nordic Red [64] and retained placenta in Canadian Holstein dairy cows [65]. *NMNAT1* was found to affect age at first calving in Nellore cattle [66], and this gene was also implicated in influencing the female reproductive efficiency in cattle [67]. In addition, we observed one gene named *CPLX1* to be related to recombination features in multiple cattle breeds [68]. *CPLX1* has been reported to be associated with the recombination rate in human and cattle studies, respectively [69,70].

Many studies have investigated the regions of homozygosity and their impacts on complex traits in humans [20,21,22,71]. For instance, several methods have been utilized to explore the association between ROH region and complex diseases [16,49,72,73]. In the present study, we utilized the region-based approach to investigate the candidate ROH regions associated with important traits in cattle, and we totally obtained 280 regions by merging all individual ROH events with the population. The proportion of ROH coverage varied for each individual, which may reflect the potential dosage effect of homozygosity for traits. Therefore, the association test was conducted between the proportion of ROH coverage and traits. We identified nine significant ROH regions, corresponding to 154 redundant genes. However, several regions contain many genes due to their large sizes, and it is difficult to pinpoint the precise site of associated ROH for studied traits. Therefore, a further assessment is warranted to attain reasonable statistical power by using sufficient sample numbers and developing new statistical approach.

## 5. Conclusions

Our study investigated the homozygosity patterns and population inbreeding level in Chinese Wagyu beef cattle. Our findings suggested that some candidate ROH regions may have potential impacts on performance for an important trait in beef cattle.

## Figures and Tables

**Table 1:** Summary of the identified ROHs and their overlapping candidate quantitative trait loci (QTLs) and related traits. The consensus ROH across the population were sorted by the frequency of ROH, and only ROH regions overlapped with QTLs (frequency exceeding 10%) were listed.

BTA	ROH-BP1	ROH-BP2	Frequency	QTL-Trait
3	54343407	54587989	0.103896	Muscularity
4	45165956	45671950	0.119048	Body weight
5	41528650	41751952	0.101732	Body weight gain
5	58882448	59612173	0.214286	Gestation length
6	33396376	33893065	0.132035	Body weight
6	80611486	80829499	0.116883	Calving ease, facial pigmentation
6	108950251	109844160	0.114719	Average daily gain, body depth, rump width, calving ease, stillbirth, Stature, strength
9	20006714	20523719	0.134199	Scrotal circumference
12	25997505	26782472	0.119048	Lean meat yield
16	43814264	44664782	0.132035	Subcutaneous fat
23	578821	1017051	0.38961	Calving ease, stillbirth

**Table 2:** Summary of the associated ROH regions for important traits using a region-based association analysis.

Chr	ROH_Left	ROH_Right	Length	ROH Count	Frequency	*p*-Value	*q*-Value	Traits	Count of Genes within ROH Region
7	12040557	15624148	3583591	33	0.071429	0.00835	0.6002	Body height	56
8	75872649	77763089	1890440	33	0.071429	0.000273	0.0765	Chest circumference	33
9	19317920	21949240	2631320	106	0.229437	0.001418	0.1985	Carcass length	4
12	39693710	41835914	2142204	95	0.205628	0.001376	0.3851	Ribeye area	0
23	7177811	8413638	1235827	6	0.012987	0.000394	0.0995	Body height	34
23	7177811	8413638	1235827	6	0.012987	0.000171	0.0480	Carcass length	34
27	36930734	37557668	626934	3	0.006494	0.000614	0.1564	Backfat thickness	9
27	13977424	15257628	1280204	5	0.010823	0.009542	0.8906	Carcass length	13
28	40886907	41889675	1002768	4	0.008658	0.001264	0.3540	Fat coverage	4

## Supplementary Materials

The following are available online at https://www.mdpi.com/2076-2615/10/8/1425/s1. Table S1: The occurrence of the consensus ROH in Chinese Wagyu beef cattle, and the consensus ROH were estimated using the PLINK command (–homozyg -group) across animals; Table S2: Summary of statistics of the ROH region including chromosome, start, end, and frequency, average of length of each ROH, and average of SNP count within each region. The ROH region was generated by aggregating overlapping CNVs (by at least 1 bp) across samples using Bedtools; Figure S1: (a) The distribution of lengths of ROH in Chinese Wagyu beef cattle, (b) the distribution of total counts of ROH across chromosomes.

## Abbreviations

The following abbreviations are used in this manuscript: SNP: Single nucleotide polymorphism; ROH: Runs of homozygosity; BTA: *Bos taurus* autosomes; MAF: Minor allele frequency; QTL: Quantitative trait loci; LD: Linkage disequilibrium; IBD: Identity by descent; GO: Gene ontology; DAVID: The Database for Annotation, Visualization, and Integrated Discovery.
