# Supplementary figures and images for: Genome-Wide Assessment of Runs of Homozygosity in Chinese Wagyu Beef Cattle
Source: Animals (Basel). 2020 Aug 14;10(8):1425. doi: 10.3390/ani10081425 (PMC7460448; doi:10.3390/ani10081425)

a

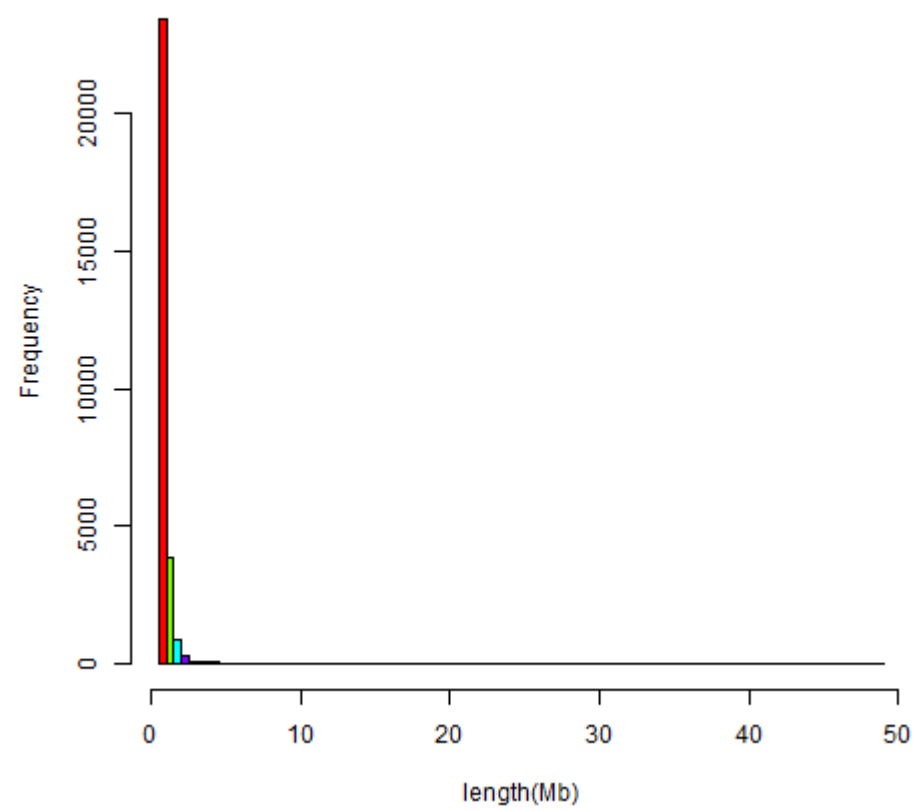

b

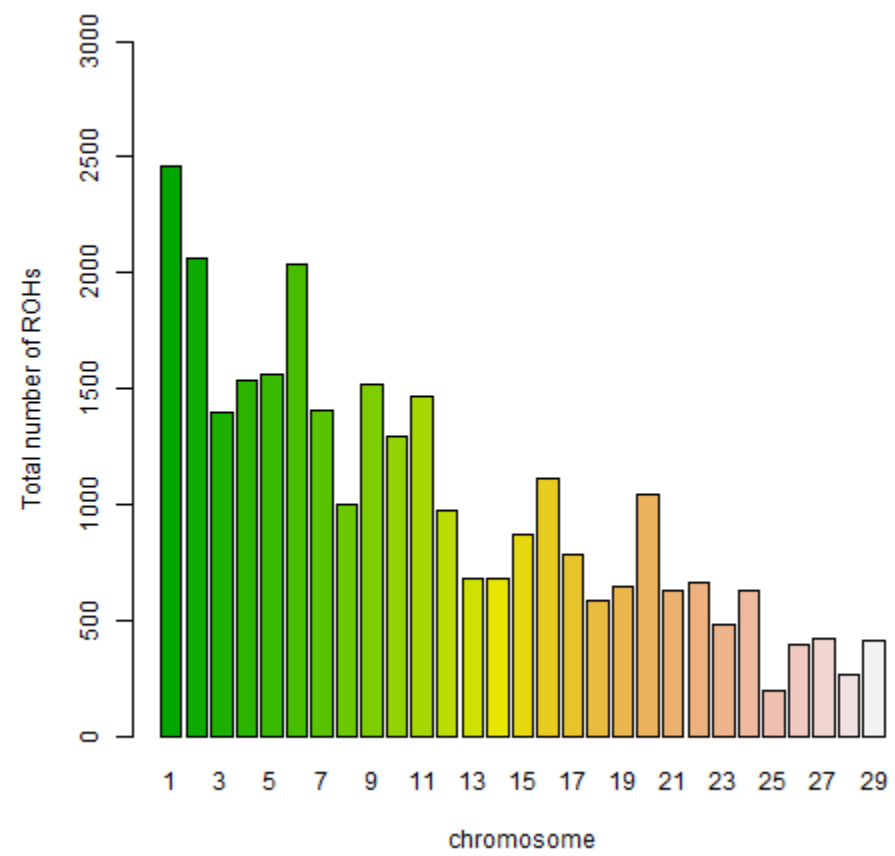

Supplement: Supplementary file 1 [file animals-10-01425-s001.zip › Supplementary Files/Figure S1.pdf]
